# The successful use of rituximab in IgA nephropathy patients with podocytopathy: a case series

**DOI:** 10.1093/ckj/sfae178

**Published:** 2024-06-27

**Authors:** Mingfang Sun, Ling Wang, Xinghong Liu, Fei Xiao, Huanzi Dai

**Affiliations:** Department of Rheumatology & Clinical Immunology, Daping Hospital & Research Institute of Surgery, Army Medical University, Chongqing, PR China; Department of Rheumatology & Clinical Immunology, Daping Hospital & Research Institute of Surgery, Army Medical University, Chongqing, PR China; Department of Nephrology, Daping Hospital & Research Institute of Surgery, Army Medical University, Chongqing, PR China; Department of Nephrology, Daping Hospital & Research Institute of Surgery, Army Medical University, Chongqing, PR China; Department of Rheumatology & Clinical Immunology, Daping Hospital & Research Institute of Surgery, Army Medical University, Chongqing, PR China

**Keywords:** IgA nephropathy, podocytopathy, rituximab

## Abstract

**Background:**

Immunoglobulin A nephropathy (IgAN) with podocytopathy is a rare pathological type of glomerular disease. The use of rituximab (RTX) in the treatment of glomerular diseases has increased in recent decades, but the efficacy of RTX in the treatment of patients with IgAN and podocytopathy has rarely been reported.

**Methods:**

This was a single-centre retrospective study of IgAN patients with podocytopathy who were treated with RTX as second-line therapy was conducted at our centre from 2019 to 2022. The aim of this study was to investigate the efficacy and safety of RTX in IgAN patients with podocytopathy.

**Results:**

Seven out of eight patients met the criteria for complete remission following RTX therapy. Only one patient experienced adverse events (infectious diarrhoea and pulmonary infection) and experienced relapse 6 months after RTX therapy. The maximum relapse-free time after RTX therapy was 20 months, while the maximum relapse-free time before RTX therapy was only 6 months. The number of relapses before RTX therapy (per year) was one to four; moreover, seven patients did not relapse and maintained remission at the last follow-up despite steroid withdrawal after RTX therapy.

**Conclusion:**

Overall, RTX effectively reduced proteinuria, increased the maximum relapse-free time, reduced the number of relapses per year and helped patients stop steroid use as soon as possible. RTX also helped most patients achieve clinical remission. RTX appears to be an effective and safe alternative for treating IgAN patients with podocytopathy with steroid dependence or frequent relapse.

KEY LEARNING POINTS **What was known:**

- The clinical role of glucocorticoids in immunoglobulin A nephropathy (IgAN) has not been fully determined.
- At present, it is recommended that IgAN patients with podocytopathy be treated in accordance with the guidelines for minimal change disease (MCD). In MCD patients, rituximab (RTX) is recommended as a second-line treatment for steroid-dependent patients or patients who experience frequent relapse.
- The efficacy and safety of RTX in IgAN patients with podocytopathy are unknown.
- We supposed to investigate the efficacy and safety of RTX in IgAN patients with podocytopathy.

**This study adds:**

- In our study, we showed that RTX treatment may have contributed to reduced proteinuria, increased the maximum relapse-free time, reduced the frequency of relapses and helped IgAN patients with podocytopathy stop steroid use as soon as possible.

**Potential impact:**

- RTX appears to be an effective and safe alternative for treating IgAN patients with podocytopathy with steroid dependence or frequent relapse.
- Further research is needed to confirm this issue and to provide the basis for the formulation of follow-up medical measures.

## INTRODUCTION

Immunoglobulin A nephropathy (IgAN) is the most common primary glomerulonephritis in children and adolescents worldwide. The clinical manifestations, pathological types and prognoses of IgAN are heterogeneous and include a wide range of symptoms and signs, from haematuria or proteinuria (usually mild to moderate proteinuria) to severe hypertension due to renal failure or acute nephritis, with rapid loss of renal function [1]. Over the last few decades, it has become clear that 10%–30% of patients who have been definitively diagnosed with IgAN by renal biopsy progress to end-stage kidney disease within 10 years after diagnosis [2]. Mesangial polymeric IgA deposition is the main pathogenetic mechanism of IgAN. At present, the management of IgAN is focused on non-immunosuppressive-based strategies, so-called supportive care, to slow the rate of disease progression. This encompasses rigorous blood pressure control, optimal inhibition by renin–angiotensin system inhibitors and lifestyle modifications, including weight reduction, exercise, smoking cessation and dietary sodium restriction. Unlike the majority of other glomerular diseases, the clinical role of glucocorticoids in IgAN has not been fully determined, and for recommended immunosuppressants (such as mycophenolate and hydroxychloroquine), only small-sample studies have shown that they may be effective.

In some cases, patients with IgAN may present with nephrotic syndrome (including oedema and both hypoalbuminaemia and nephrotic-range proteinuria >3.5 g/day). Mesangial IgA deposition can be associated with light and electron microscopy features that are otherwise consistent with podocytopathy resembling minimal change disease (MCD). It is unclear whether nephrotic syndrome is a specific podocytopathic variant of IgAN or indicates the presence of mesangial IgA deposition in MCD patients. In these cases, several scholars believe that this is a special type of podocytosis, so-called IgAN with podocytopathy, rather than typical IgAN. Several associations between IgAN and other glomerular diseases have already been reported; the association between IgAN and focal segmental glomerulosclerotic lesions has been extensively described [3–6] and represents an important negative prognostic element in disease progression. However, correlational research comparing IgAN and primary podocytopathy is rare [7].

Podocyte injury is a factor underlying the development of proteinuria and glomerulopathies and has been used as a clinical prognostic factor for IgAN [8, 9]. Glomerulosclerotic lesions, usually tip lesions, are relatively frequent in IgAN patients and are usually a sign of secondary glomerulosclerosis caused by a critical reduction in the nephron population and related glomerular hyperfiltration. The clinical course of proteinuria in these patients is usually mild, and chronic lesions are usually evident in kidney biopsies. Furthermore, a response to immunosuppressive treatment is usually absent. Conversely, primary podocyte injury is characterized by a rapid and difficult clinical course with severe proteinuria, and these patients often respond quickly to immunosuppressive treatment. At present, it is recommended that IgAN patients with podocytopathy be treated in accordance with the guidelines for MCD, which is usually treated with glucocorticoids and immunosuppressive therapy rather than supportive care.

Rituximab (RTX) was first approved for clinical use in the late 1990s and has since been one of the most commonly used and important medications in clinical practice [10]. It is most commonly used for the treatment of haematologic malignancies, although its use in patients with autoimmune diseases has been increasing [10]. As a monoclonal antibody that targets CD20 on B cells, RTX has been used, at least somewhat successfully, for a variety of conditions mediated by B cells, including rheumatoid arthritis, Sjögren's syndrome systemic lupus erythematosus, antineutrophilic cytoplasmic antibody–associated vasculitis and many types of nephropathy [11–16]. As is the case with cytotoxic drugs, RTX reduces the necessary dosage of corticosteroids and decreases the likelihood of side effects associated with corticosteroid use [17]. Compared with most cytotoxic drugs, RTX is generally well tolerated, although adverse reactions, including serious reactions, have been reported (e.g. acute respiratory distress syndrome, myocardial infarction and toxic epidermal necrolysis).

RTX has been used to treat IgAN, although the international guideline recommendations for its use have not been clearly defined. Several clinical trials with small sample sizes suggested that RTX might be effective in the treatment of rapidly progressive glomerulonephritis caused by IgAN. In MCD patients, RTX is recommended as a second-line treatment for steroid-dependent patients or patients who experience frequent relapse [18]. In this article, we present a single-centre experience with the use of RTX for the treatment of IgAN patients with podocytopathy in terms of clinical presentation, immunosuppressant protocol, outcomes and complications, and provide a narrative review of the efficacy and safety of this therapy.

## MATERIALS AND METHODS

### Case reports

#### Patient 1

Patient 1 was a 16-year-old male who was steroid dependent but unable to taper off prednisone, and relapse occurred each time the dose was reduced to five tablets. His creatinine, albumin and proteinuria levels were 42.8 µmol/L, 15.1 g/L and 5.73 g/day, respectively. He received one course of RTX 375 mg/m^2^ weekly for 4 weeks and remained relapse-free at the last follow-up, with no other immunosuppressive agent used for 8 months.

#### Patient 2

Patient 2 was a 17-year-old male for whom combination therapy with prednisone and tripterygium glycosides had previously failed. His creatinine, albumin and proteinuria levels were 47.1 µmol/L, 16.1 g/L and 8.48 g/day, respectively. He was treated with 375 mg/m^2^ RTX weekly for 4 weeks, followed by a single 375 mg/m^2^ dose with CD20 reconstitution (approximately 7 months later). Three months after the first dose of RTX, the patient was tapered off all other immunosuppressive agents and remained in complete remission (CR), which was defined as 24-h proteinuria level <0.3 g/24 h.

#### Patient 3

Patient 3 was a 16-year-old male who was steroid-dependent but unable to taper off prednisone without a disease flare. His creatinine, albumin and proteinuria levels were 52.5 µmol/L, 37.3 g/L and 0.94 g/day, respectively. He was treated with only one dose of 375 mg/m^2^ RTX. RTX was not used for economic reasons. He achieved CR 1 month after his first RTX dose. Two months after the initiation of RTX therapy, the patient was tapered off all other immunosuppressive agents and remained in CR at the last follow-up.

#### Patient 4

A 25-year-old female was referred for IgAN with podocytopathy. She was steroid dependent, but she experienced frequent relapse (twice per year, and the maximum relapse-free time was 3 months), even with the combined use of steroids and tacrolimus. She received 375 mg/m^2^ RTX weekly for 4 weeks and remained relapse-free at the last follow-up without the use of any other immunosuppressive agents for 14 months.

#### Patient 5

Patient 5 was diagnosed with IgAN with podocytopathy at 23 years of age. He experienced frequent relapse (twice per year, and the maximum relapse-free time was 5 months) and received one course of RTX 375 mg/m^2^ weekly for 4 weeks. Before RTX therapy, the patient's maximum relapse-free time was 5 months, and after infusion, he remained relapse-free at the last follow-up without the use of any other immunosuppressive agents for 9 months.

#### Patient 6

Patient 6 was a 54-year-old male who was steroid dependent. After treatment with steroids, the patient achieved CR and did not relapse during the first 3 months; however, he was diagnosed with steroid-induced diabetes mellitus. He refused to continue taking steroids and asked for a change in treatment. The patient was treated with two doses of RTX (375 mg/m^2^). Afterwards, he stopped steroid use as soon as possible, and he became steroid-free 1 month after RTX therapy.

#### Patient 7

Patient 7 was diagnosed with IgAN at 19 years of age. He experienced frequent relapses (twice per year, and the maximum relapse-free time was 4 months). He received one course of RTX 375 mg/m^2^ weekly for 4 weeks and remained relapse-free at the last follow-up without the use of any other immunosuppressive agents for 8 months.

#### Patient 8

Patient 8 was a 14-year-old male who was steroid-dependent but unable to taper prednisone, which was defined as steroid dependency. He received two courses of RTX 375 mg/m^2^ and achieved CR, quickly becoming steroid-free 2 months after RTX therapy. Unfortunately, 6 months after the first infusion, he developed infectious diarrhoea and pulmonary infection and experienced full relapse. His urinary albumin–creatinine ratio rapidly increased to 5170.57 mg/g. Due to the probable side effects of RTX, he refused to continue the treatment.

### Ethics statement

The studies involving humans were approved by the Ethics Committee of Daping Hospital, Army Medical University. The studies were conducted in accordance with the local legislation and institutional requirements. The participants provided their written informed consent to participate in this study. Written informed consent was obtained from the individual(s) for the publication of any potentially identifiable images or data included in this article.

## RESULTS

**Figure 1:**
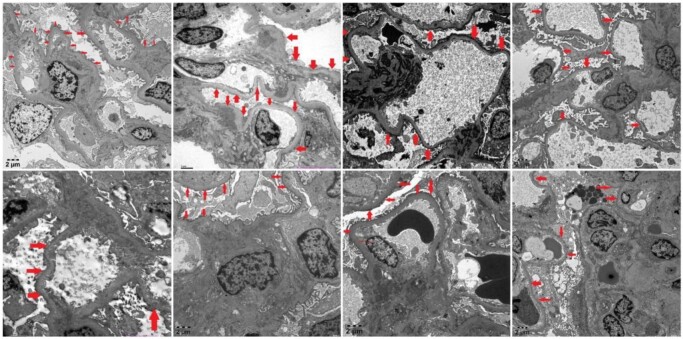
The typical electron microscopy images of patients 1–8.

**Figure 2:**
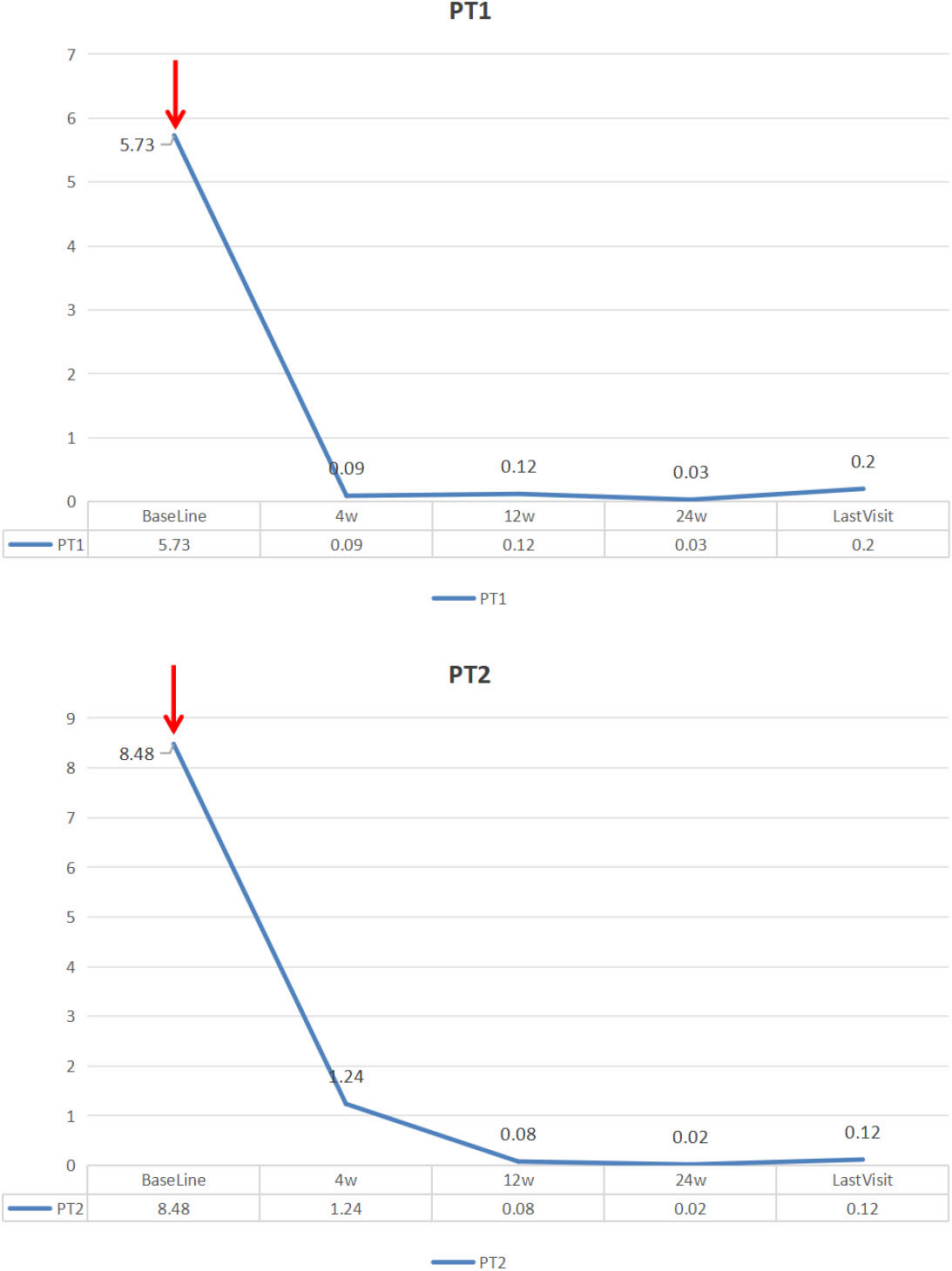

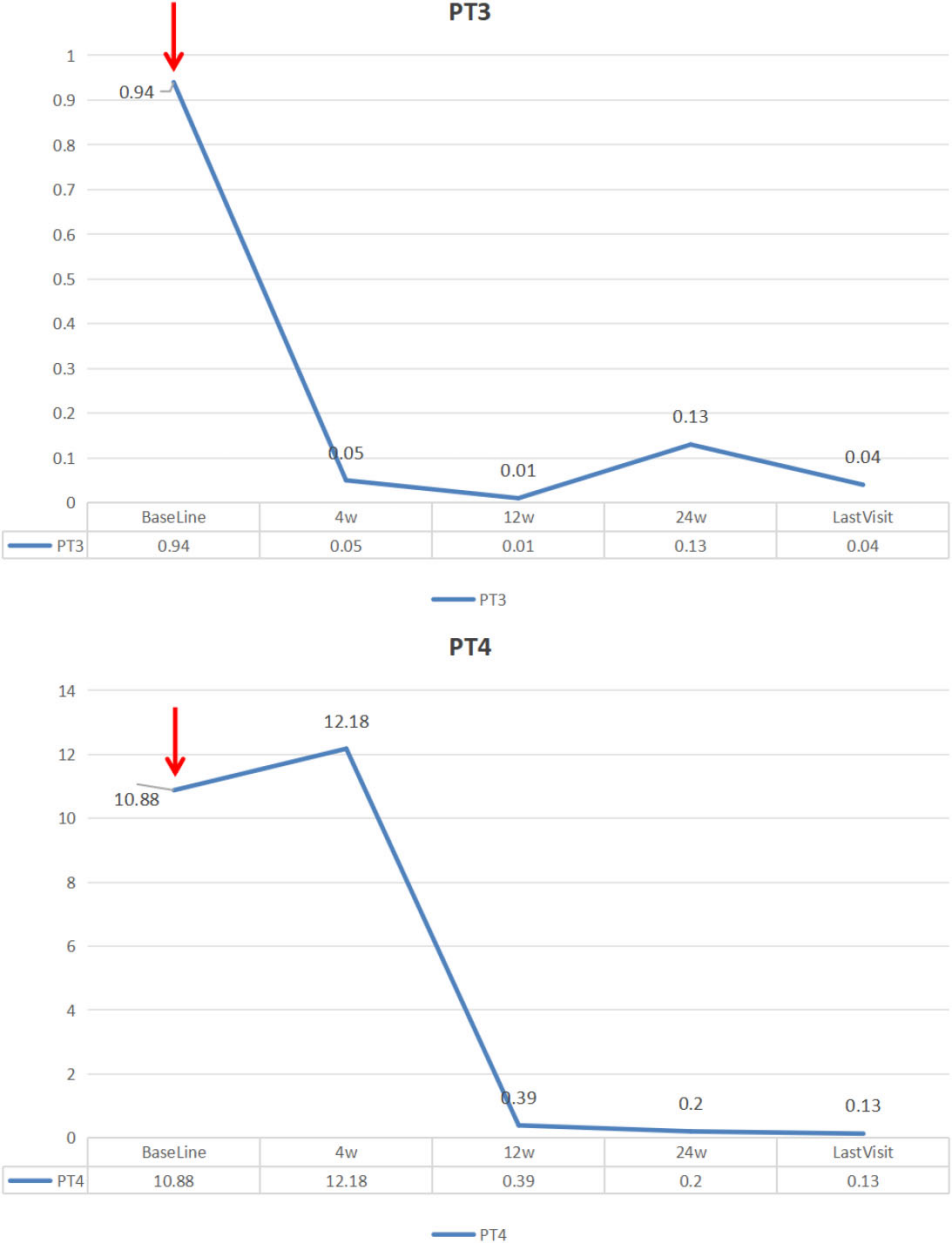

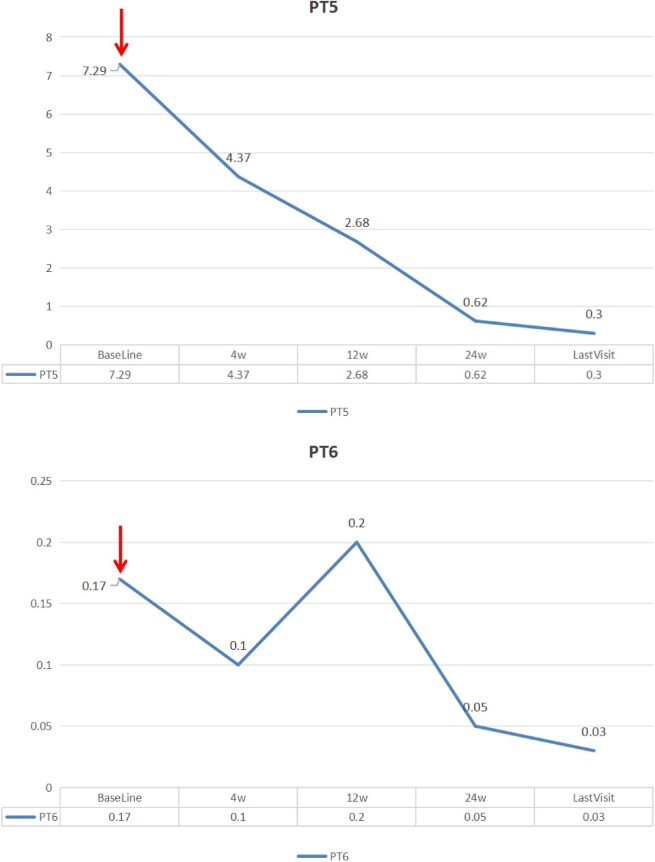

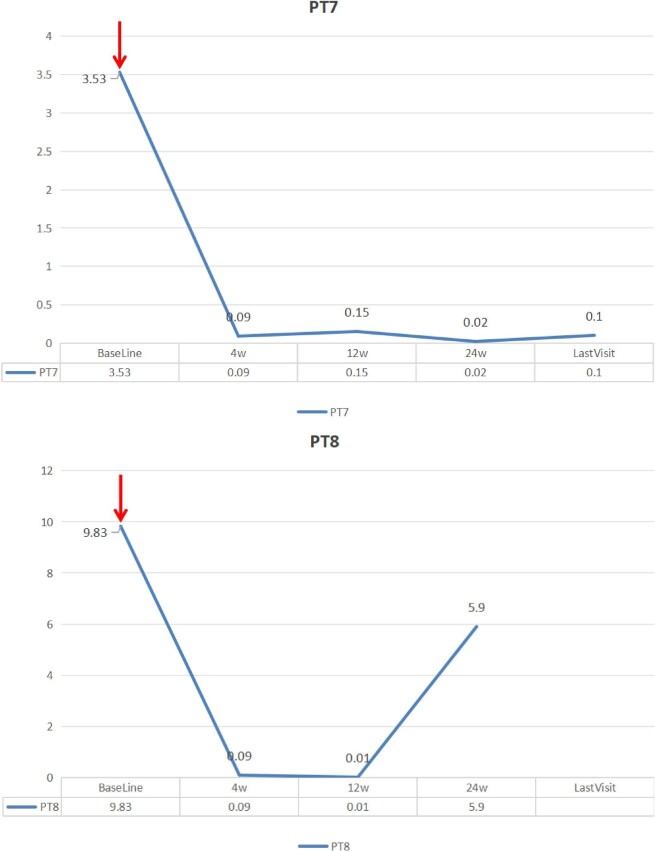
The changes in 24-h proteinuria of patients 1–8. Red arrow: The first time of RTX infusion.

Overall, eight patients were included in the study. All patients underwent kidney biopsy and electron microscopy. The basic pathological manifestations of these patients were as follows: IgA deposition could be detected in the mesangial area via immunofluorescence, and mild lesions could be detected via light microscopy. Under an electron microscope, in addition to the deposition of electron-dense matter in the mesangial area, this process is often accompanied by ‘foot process extensive fusion’. Typical electron microscopy images of these patients are shown in Fig. 1. These patients showed promising responses to treatment with RTX. There were seven males and one female (aged 14–54 years at disease onset), and their clinical characteristics at baseline are shown in Table 1. The changes in 24-h proteinuria in patients 1–8 are illustrated in Fig. 2. These patients exhibited 24-h proteinuria at baseline (the time at which RTX was first infused), 4 weeks after the first infusion of RTX, 12 weeks after the first infusion, 24 weeks after the first infusion and until the last visit.

**Table 1:** Patient clinical characteristics at baseline (the time at which RTX was first infused).

	Patient 1	Patient 2	Patient 3	Patient 4	Patient 5	Patient 6	Patient 7	Patient 8
Age at diagnosis (years)	16	17	16	25	23	54	19	14
Gender	M	M	M	F	M	M	M	M
BMI	18.5	22.2	16	26.6	25.1	22.1	22.2	20.5
Biopsy time^a^ (month)	9	21	16	25	7	3	10	4
eGFR (mL/min/1.73 m^2^)	161.1	155.2	147.1	122.46	138.83	52.42	149.71	135.91
Serum albumin (g/L)	15.1	16.1	37.3	28.7	15.7	40.7	18.0	18.9
24 h urinary protein (g/24 h)	5.73	8.48	0.94	10.88	7.29	0.17	3.53	9.83
IgA (g/L)	1.87	1.4	1.96	3.07	2.78	1.88	0.68	2.04
Urinary albumin–creatinine ratio (mg/g)	4035.09	4851.34	988.92	7811.37	4850.31	82.4	6236.98	9304.92
Serum creatinine (µmol/L)	42.8	47.1	52.5	55.7	53.9	131.8	49.7	68.5
Serum cholesterol (mmol/L)	10.15	11.41	7.21	6.47	6.64	4.91	16.4	8.96
Serum triglycerides (mmol/L)	1.12	4.73	0.91	4.28	1.85	1.23	0.69	1.76
HDL cholesterol (mmol/L)	4.47	1.86	1.34	1.26	1.29	1.21	4.58	2.24
LDL cholesterol (mmol/L)	5.12	6.44	4.82	3.82	4.12	2.98	8.94	5.58

^a^The time from kidney biopsy to the first infusion of RTX.

M: male; F: female; BMI: body mass index; HDL: high-density lipoprotein; LDL: low-density lipoprotein.

At presentation, the average serum creatinine level was 62.75 (42.8–131.8) µmol/L, the baseline 24-h urine protein level was 5.86 (0.17–10.88) g and the mean serum albumin level was 23.81 g/L (15.1–40.7). One patient had an elevated serum creatinine level. The mean estimated glomerular filtration rate (eGFR) was 132.84 mL/min per 1.73 m^2^ (52.42–161.1). All patients received steroid treatment at disease onset. Five patients had steroid dependency, and two patients had frequent relapses. For the one patient who was diagnosed with steroid-induced diabetes mellitus after the use of steroids, RTX treatment was provided.

After the first infusion of RTX, seven patients achieved CR by the last follow-up, and one patient experienced relapse 6 months after RTX therapy. The maximum relapse time after RTX therapy was 20 months, while the maximum relapse-free time before RTX therapy was only 6 months. The number of relapses (per year) before RTX therapy was one to four; however, seven patients did not experience relapse and maintained remission at the last follow-up despite steroid withdrawal after RTX therapy (Table 2). The cumulative RTX dose, treatment response before RTX, follow-up after RTX therapy, steroid-free status after RTX treatment, maximum relapse-free time before RTX treatment, maximum relapse-free time after RTX treatment, number of relapses before RTX treatment and number of relapses after RTX treatment are illustrated in Table 2.

**Table 2:** Clinical data of the follow-up.

	Renal biopsy pathology	Reasons for usage	Previous immunosuppressant	Cumulative RTX dose (mg)	Response before RTX	Status at last follow-up	Follow-up after RTX therapy (months)	Steroid free after RTX (months)	Max relapse free before RTX (months)	Max relapse free after RTX (month)	Number of relapses before RTX (per year)	Number of relapses after RTX (per year)	Complications of RTX therapy
PT1	M1E1S1C0TO	SD	CST	2400	FR	CR	8	2	2	8	2	0	None
PT2	M1E0S0C0T0	SD	CST, TG	3000	FR	CR	20	3	6	20	4	0	None
PT3	M1E1S0C0T0	SD	CST	600	PR	CR	10	2	4	10	4	0	None
PT4	M1E0S1C0T0	FR	CST, TAC	2400	FR	CR	14	2	3	14	2	0	None
PT5	M1E0S1C0T0	FR	CST	2400	FR	CR	9	5	5	9	2	0	None
PT6	M1E1S1C0T0	SDM	CST	1200	CR	CR	8	1	/	8	/	0	None
PT7	M1E1S1C2T0	SD	CST	2400	FR	CR	8	4	4	8	2	0	None
PT8	M1E1S1C0T0	SD	CST	1200	PR	FR	9	2	2	6	1	1	Infectious diarrhoea and pulmonary infection

PT: patient; SDM: steroid-induced diabetes mellitus; FR: frequent relapse; SD: steroid dependency; CST: corticosteroids; PR: partial remission; CR: complete remission; TG: tripterygium glycosides; TAC: tacrolimus.

### Adverse effects

Seven patients tolerated RTX treatment without any adverse effects. One patient experienced infectious diarrhoea and pulmonary infection 6 months after the first infusion and experienced full relapse. After a course of intravenous and oral antibiotics, he recovered and restarted induced remission therapy with adequate prednisone.

## DISCUSSION

In this case series, we investigated the efficacy and safety of RTX treatment in refractory IgAN patients with podocytopathy who had experienced steroid dependency or relapse after treatment with other immunosuppressive agents. All patients (seven out of eight) who received RTX treatment achieved nearly CR at the last follow-up, with no significant safety issues.

At present, the academic opinion of the pathogenesis of IgAN considers the four-hit theory: a defect in the regulation of IgA1 production and glycosylation (Hit 1), anti-glycan antibodies (Hit 2), immune complex formation and deposition (Hit 3) and local activation of inflammatory pathways and the complement system (Hit 4) [19]. IgAN is an autoimmune disease characterized by the deposition of pathogenic IgA1 immune complexes in the glomerular mesangium, which then activates mesangial cells and the immune system, inducing renal injury. A critical characteristic of IgAN is immune complexes containing galactose-deficient (Gd)-IgA1, which highlights the pathogenic role of B cells.

The clinical course of proteinuria in patients with IgAN is usually mild (mild to moderate), and chronic lesions are usually evident in kidney biopsies. Therefore, a response to immunosuppressive treatment is usually absent. Conversely, primary podocyte injury is characterized by a rapid and difficult clinical course with severe proteinuria, and these patients often respond quickly to immunosuppressive treatment. Podocytopathy in IgAN patients can result from inflammatory mediators, including complement activation products, reactive oxygen species and cytokines released into the circulation or, most likely, in the glomerular area during the inflammatory process following the deposition of IgA-containing immune complexes. This might bring about an acute condition driven by endocapillary hypercellularity or continuous cross talk between mesangial cells and podocytes [7, 20]. In the first case, proteinuria can develop suddenly and disappear after rapid reversal of acute endocapillary lesions. The second mechanism is likely to occur more chronically, mostly through mesangial cell reactivity and the induction of clinically evident functional damage, such as proteinuria. In cultured mesangial cells, aberrantly glycosylated IgA1, defective in galactose (Gd-IgA1) and macromolecular IgA isolated from patients with IgAN were found to trigger the production of tumour necrosis factor alpha and other cytokines that synergistically act on podocytes, activating the nuclear translocation of nuclear factor kappa B and reducing the expression of nephrin and ezrin, which are pivotal proteins involved in podocyte-regulated slit diaphragm physiology [21, 22].

As we mentioned in the Introduction, it is recommended that IgAN patients with podocytopathy be treated in accordance with the guidelines for MCD, which is usually treated with glucocorticoids and immunosuppressive agents, with over 80% of patients achieving remission. However, MCD is a relapsing disease. Most patients relapse infrequently after remission, but a significant minority of patients relapse frequently or become dependent on steroids. Several clinical trials have suggested that RTX may be considered an additional treatment for MCD patients [23]. However, it remains unclear how RTX leads to remission in patients with MCD. Recently, the discovery of autoantibodies targeting nephrin in MCD patients supported a novel autoimmune aetiology [24]. Andrew *et al*. reported that the levels of circulating nephrin autoantibodies during active disease were significantly reduced or absent during treatment in a subset of patients with MCD. Another pilot study of anti-nephrin antibodies in podocytopaties among adults showed that the level of anti-nephrin antibodies was significantly greater in patients with MCD, indicating that anti-nephrin antibodies in MCD may be associated with the severity of podocytopathies [25]. These findings may provide a possible basis for the efficacy of RTX in patients with refractory and relapsing MCD. There is no doubt that targeted B-cell therapy is critical. In addition, RTX has also been shown to play roles in B-cell-independent mechanisms. For instance, RTX was demonstrated to regulate the activity of acid-sphyngomyelinase (ASMase), which is essential for signalling molecules on podocytes [26]. Perosa *et al*. reported that RTX might cross-react with sphingomyelin-phosphodiesterase-acid-like-3b (SMPDL-3b) [27]. Rather than directly acting on antibody production, RTX might prevent actin cytoskeleton remodelling in podocytes by preserving sphingolipid-related enzymes and SMPDL-3b and ASMase activity, thus playing a role in the protection of podocytes. Therefore, the KDIGO guidelines [18] recommend RTX for the treatment of frequently relapsing and steroid-dependent MCD patients, which can help reduce the number of relapses and improve steroid dependence. As we mentioned before, several scholars believe that IgAN with podocytopathy is a special type of podocytosis rather than typical IgAN (which may not benefit from glucocorticoids or immunosuppressants). Other researchers defined MCD-like variant IgAN with diffuse foot process fusion under an electron microscope as MCD-IgAN. In our study, IgAN patients with podocytopathy tended to respond well to RTX, which seems to be a reasonable alternative treatment agent. RTX effectively reduced proteinuria, reduced the number of relapses and helped patients stop steroid use as soon as possible. RTX also helped most patients achieve CR. RTX appears to be an effective and safe alternative for treating steroid-dependent or frequent relapse in IgAN patients with podocytopathy. This finding further confirmed that IgAN with podocytopathy is pathophysiologically different from IgAN with podocytopathy. However, further research is needed to confirm this issue.

Our observational study has several limitations. This study was retrospective and observational. The lack of power due to the small sample size in our cohort makes it difficult to draw conclusions about null findings. Moreover, the lack of a control group is also a limitation of the observational nature of the study. Steroid therapy can make it difficult to interpret the prognostic value of pathological parameters. In our study, we showed that RTX treatment may have contributed to reduced proteinuria, increased the maximum relapse-free time and reduced the number of relapses per year, and helped patients stop steroid use as soon as possible.

## Data Availability

The data that support the findings of this study are available on request from the corresponding author upon reasonable request.
